# Tyrosine as a Mechanistic-Based Biomarker for Brain Glycogen Decrease and Supercompensation With Endurance Exercise in Rats: A Metabolomics Study of Plasma

**DOI:** 10.3389/fnins.2019.00200

**Published:** 2019-03-19

**Authors:** Takashi Matsui, Yu-Fan Liu, Mariko Soya, Takeru Shima, Hideaki Soya

**Affiliations:** ^1^Laboratory of Exercise Biochemistry and Neuroendocrinology, Faculty of Health and Sport Sciences, University of Tsukuba, Tsukuba, Japan; ^2^Sport Neuroscience Division, Advanced Research Initiative for Human High Performance (ARIHHP), University of Tsukuba, Tsukuba, Japan

**Keywords:** endurance exercise, brain glycogen, supercompensation, plasma biomarker, metabolomics

## Abstract

Brain glycogen, localized in astrocytes, produces lactate as an energy source and/or a signal factor to serve neuronal functions involved in memory formation and exercise endurance. In rodents, 4 weeks of chronic moderate exercise-enhancing endurance and cognition increases brain glycogen in the hippocampus and cortex, which is an adaption of brain metabolism achieved through exercise. Although this brain adaptation is likely induced due to the accumulation of acute endurance exercise–induced brain glycogen supercompensation, its molecular mechanisms and biomarkers are unidentified. Since noradrenaline synthesized from blood-borne tyrosine activates not only glycogenolysis but also glycogenesis in astrocytes, we hypothesized that blood tyrosine is a mechanistic-based biomarker of acute exercise–induced brain glycogen supercompensation. To test this hypothesis, we used a rat model of endurance exercise, a microwave irradiation for accurate detection of glycogen in the brain (the cortex, hippocampus, and hypothalamus), and capillary electrophoresis mass spectrometry–based metabolomics to observe the comprehensive metabolic profile of the blood. Endurance exercise induced fatigue factors such as a decrease in blood glucose, an increase in blood lactate, and the depletion of muscle glycogen, but those parameters recovered to basal levels within 6 h after exercise. Brain glycogen decreased during endurance exercise and showed supercompensation within 6 h after exercise. Metabolomics detected 186 metabolites in the plasma, and 110 metabolites changed significantly during and following exhaustive exercise. Brain glycogen levels correlated negatively with plasma glycogenic amino acids (serine, proline, threonine, glutamate, methionine, tyrosine, and tryptophan) (*r* < −0.9). This is the first study to produce a broad picture of plasma metabolite changes due to endurance exercise–induced brain glycogen supercompensation. Our findings suggest that plasma glycogenic amino acids are sensitive indicators of brain glycogen levels in endurance exercise. In particular, plasma tyrosine as a precursor of brain noradrenaline might be a valuable mechanistic-based biomarker to predict brain glycogen dynamics in endurance exercise.

## Introduction

Brain glycogen localized in astrocytes plays the role of an energy source and/or signaling factor in maintaining neuronal functions, such as memory formation and exercise endurance (Suzuki et al., 2011; Matsui et al., 2017; Magistretti and Allaman, 2018). Chronic exercise enhances memory functions and endurance capacity as well as raises the glycogen levels of the cortex and hippocampus in healthy and type II diabetic rats (Matsui et al., 2012; Shima et al., 2017). This metabolic adaptation of the brain, due to chronic exercise, should be induced by the accumulation of brain glycogen supercompensation after an acute exercise-induced decrease in brain glycogen (Matsui et al., 2011, 2012, 2017). Therefore, brain glycogen dynamics during and following exercise can be a valuable parameter for exercises as training/conditioning for athletes and/or a therapeutic strategy for neurodegenerative diseases.

To date, however, the understanding of human brain glycogen metabolism is still less clear. A previous study using biopsy samples reported that hippocampal glycogen levels were higher compared to other brain regions in patients with epilepsy (Dalsgaard et al., 2007). However, since brain biopsy procedures carry the risk of parenchymal hemorrhage, it is difficult to use in healthy or vulnerable people (Beynon et al., 2018). Although a non-invasive measurement for brain glycogen metabolism has been developed using *in vivo* nuclear magnetic resonance (NMR) in healthy people and type I diabetes patients (Oz et al., 2009, 2017), human brain glycogen metabolism during exercise remains unclear because the head movement can cause noise preventing the accurate measurement of brain glycogen using *in vivo* NMR (Oz et al., 2009). To resolve this issue, non-invasive identification of biomarkers that can predict brain glycogen dynamics with exercise is desirable.

Interestingly, in astrocytes, noradrenaline synthesized from blood-borne tyrosine, activates glycogenolysis through cyclic adenosine monophosphate (cAMP) production in a matter of minutes (Magistretti et al., 1981), but takes hours to induce glycogen synthesis and supercompensation through the expression of protein targeting to glycogen (PTG) (Sorg and Magistretti, 1992; Allaman et al., 2000; Ruchti et al., 2016). We have previously reported that acute endurance exercise decreases brain glycogen levels associated with increased brain tyrosine and noradrenaline levels (Matsui et al., 2011, 2017). We thus hypothesized that blood tyrosine is a mechanistic-based biomarker for the decrease and supercompensation of brain glycogen with acute endurance exercise.

To test this hypothesis, we employed a rat model of acute endurance exercise, high-power microwave irradiation for accurate detection of brain glycogen (10 kW) (Kong et al., 2002; Matsui et al., 2011), and plasma metabolomics using capillary electrophoresis-mass spectrometry (CE-MS). Biomarkers for various disorders, including various types of cancers, have been identified in previous studies using metabolomics, which can analyze comprehensive metabolites (Tomita and Kami, 2012). Its utility has been demonstrated by identifying new biomarkers for prostate cancer (Sreekumar et al., 2009), Parkinson’s disease (Bogdanov et al., 2008), type 2 diabetes mellitus (Wang et al., 2005), acute myocardial ischemia (Sabatine et al., 2005), and non-alcoholic fatty liver disease in humans and rodents (Soga et al., 2006, 2011).

## Materials and Methods

### Animals

Adult male Wistar rats (250–300 g) (SLC Inc., Shizuoka, Japan), housed and cared for in an animal facility, were fed a standard pellet diet (MF, Oriental Yeast Co., Ltd., Tokyo, Japan) and given water *ad libitum*. The room temperature was maintained between 22 and 24°C under a 12 h light–12 h dark cycle (lights on: 0700–1900). Fifteen rats were habituated to run on a treadmill (SN-460, Shinano, Tokyo, Japan) for five sessions over 6 days, 30 min/day. The running speed was gradually increased from 5 to 25 m/min (see Supplementary Table S1; Matsui et al., 2011, 2012, 2017; Nishijima et al., 2012).

### Surgery

Surgery was performed according to methods described by Soya et al. (2007). After habituation to treadmill running, the rats were anesthetized with isoflurane, and a silicone catheter was inserted into the jugular vein and fixed with a silk thread (32 mm). The external distal end of the catheter was fixed at the animal’s nape.

### Endurance Exercise

Two days after surgery, rats were fasted for 2 h before exercise to obtain stable metabolic conditions. They were exercised to exhaustion on a treadmill at 20 m/min, which is defined as moderate intensity around the lactate threshold in the rat mode of exercise (Ohiwa et al., 2006; Soya et al., 2007; Okamoto et al., 2012; Inoue et al., 2015; Shima et al., 2017). Exhaustion was considered to be achieved when the rat was no longer able to keep pace with the treadmill and when the rat laid flat on the treadmill, and stayed on the grid positioned at the back of the treadmill for a period of 30 s despite being gently pushed with sticks or breathed on (Matsui et al., 2011, 2012, 2017).

### Sacrifice of Animals and Tissue Extraction

We collected tissue samples according to a previous study confirming a decrease and supercompensation of brain glycogen with exercise (Matsui et al., 2012). At the pre-exercise, immediately after exercise (post-0 h), and 6 h after exercise (post-6 h), the rats were anesthetized with isoflurane in a bell jar for less than 1 min and sacrificed using focused microwave irradiation (MI) (10 kW, 1.2 s; NJE-2603, New Japan Radio Co., Ltd., Tokyo, Japan). Previous studies have confirmed that brain glycogen levels are unchanged by this duration of isoflurane challenge (Kong et al., 2002; Matsui et al., 2011). After MI, brain tissues (cortex, hippocampus, and hypothalamus) were collected according to Hirano et al. (2006). Skeletal muscle (plantaris) and blood were also collected. All samples were stored at −80°C until analysis.

### Blood Glucose and Lactate Assays

Blood glucose and lactate levels were measured using an automated glucose-lactate analyzer (2300 Stat Plus, Yellow Springs Instruments, United States).

### Glycogen Assay

Tissues were homogenized with a bead homogenizer (MS-100R, TOMY, Tokyo, Japan) in ice-cold 6% perchloric acid (PA) containing 1 mM EDTA. The glycogen contained in 100 μl aliquots of homogenate was hydrolyzed to glucose by incubation for 3 h at 37°C with 1 ml 0.2 M sodium acetate, 20 μl 1.0 M KHCO_3_, and 20 U/ml amyloglucosidase. The addition of 0.5 ml PA stopped the reaction. After centrifugation (14,000 × *g* for 10 min at 4°C), the supernatant was neutralized with a solution consisting of 3 M KOH, 0.3 M imidazole, and 0.4 M KCl. The sample was then centrifuged (16,000 × *g* for 10 min at 4°C) and the supernatant was assayed for glucose content. Non-hydrolyzed samples were used to measure endogenous glucose levels. These samples were homogenized and centrifuged (14,000 × *g* for 10 min at 4°C), and the pH of the supernatants was adjusted to 6–8 using the KOH solution. The neutralized samples were then mixed thoroughly, centrifuged (16,000 × *g* for 10 min at 4°C), and assayed for endogenous glucose levels. The glucose assay was performed in 96-well plates using a coupled enzyme assay method modified from previous studies (Matsui et al., 2011, 2012).

### Metabolomics by Capillary Electrophoresis-Time-of-Flight Mass Spectrometry

Capillary electrophoresis-time-of-flight mass spectrometry was conducted by Human Metabolome Technologies Co., Ltd., (Yamagata, Japan) to determine the metabolomics (Sugiura et al., 2011). Each frozen sample was homogenized in methanol (500 mL/100 mg tissue) using a bead homogenizer (Micro Smash MS-100R; TOMY, Tokyo, Japan), followed by the addition of an equal volume of chloroform and 0.4 times the volume of Milli-Q water. After centrifugation (3 cycles at 5,000 × *g* for 60 s), the aqueous phases were ultrafiltered using an ultrafiltration tube (Ultrafree-MC, UFC3 LCC; Millipore, United States) and the filtrates were dried. The dried residues were redissolved in 50 mL Milli-Q water and were used for CE-MS. CE-MS experiments were performed using Agilent CE systems equipped with a time-of-flight mass spectrometer (TOF-MS) and a built-in diode-array detector (Agilent Technologies, Santa Clara, United States). Cationic metabolites were analyzed using a fused-silica capillary (50 mm i.d., 680 cm total length) with cation buffer solution (Human Metabolome Technologies) as the electrolyte. The samples were injected at a pressure of 5.0 kPa for 10 s (approximately 10 nL). The applied voltage was set at 30 kV. Electrospray ionization mass spectrometry (ESI-MS) was conducted in the positive ion mode, and the capillary voltage was set at 4,000 V. The spectrometer was scanned from m/z 50 to 1,000. Other conditions were the same as in the cation analysis (Soga and Heiger, 2000). Anionic metabolites were analyzed using a fused-silica capillary (50 mm i.d., 680 cm total length), with anion buffer solution (Human Metabolome Technologies) as the electrolyte. The samples were injected at a pressure of 5.0 kPa for 25 s (approximately 25 nL). The applied voltage was set at 30 kV. ESIMS was conducted in the negative ion mode, and the capillary voltage was set at 3,500 V. The sample in the spectrometer was scanned from m/z 50 to 1,000. Other conditions were the same as described for the anion analysis. Metabolites in the samples were identified by comparing the migration time and m/z ratio with authentic standards, and differences of 60.5 min and 610 ppm, respectively, were permitted. The identified metabolites were quantified by comparing their peak areas with those of authentic standards using ChemStation software (Agilent Technologies).

The metabolomics data were adopted for principal component analysis (PCA) and hierarchical cluster analysis (HCA) using software by Human Metabolome Technologies. Data were also visualized on a metabolome-wide pathway map for glycolysis and the TCA cycle supported by VANTED software (Junker et al., 2006).

### Statistical Analysis

Data are expressed as mean ± standard error and were analyzed using Prism 5 (MDF Co., Ltd., Tokyo, Japan). Group comparisons were performed using a one-way ANOVA with Tukey’s *post hoc* tests. Correlations were calculated using Pearson’s product-moment correlations. Statistical significance was assumed at *P*-values <0.05.

## Results

### Endurance Exercise Induces Decrease and Supercompensation in Brain Glycogen

**FIGURE 1:**
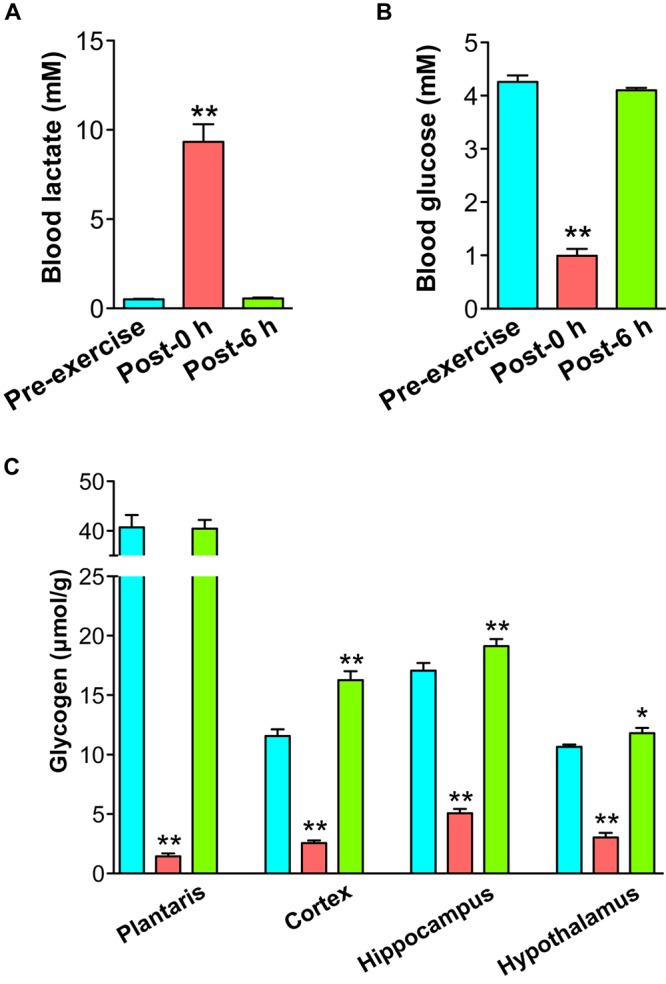
Endurance exercise induces a decrease and supercompensation in brain glycogen. **(A)** Blood lactate levels. **(B)** Blood glucose levels. **(C)** Glycogen levels in the plantaris muscle, cortex, hippocampus, and hypothalamus. Data are expressed as mean ± standard error (*n* = 5/group). ^∗^*P* < 0.05; ^∗∗^*P* < 0.01 vs. pre-exercise group.

Rats were exercised on the treadmill until exhaustion (20 m/min, time to exhaustion: 89.5 ± 5.2 min). Blood lactate was significantly increased by endurance exercise (*P* < 0.01) and recovered to basal levels within 6 h after exercise (Figure 1A). Blood glucose levels were significantly decreased by endurance exercise (*P* < 0.01) and recovered to basal levels within 6 h after exercise (Figure 1B). Muscle glycogen levels were depleted by endurance exercise (*P* < 0.01), and it recovered to basal levels within 6 h after exercise (Figure 1C). Brain glycogen levels in the cortex, hippocampus, and hypothalamus were significantly decreased by endurance exercise (*P* < 0.01), but were replenished to higher levels in comparison to the pre-exercise group (*P* < 0.05) (Figure 1C).

### Plasma Metabolomics in Exercising Rats

**FIGURE 2:**
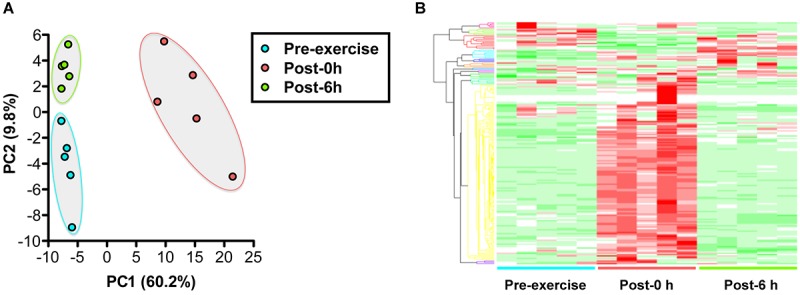
Changes in the plasma metabolic profile with endurance exercise. **(A)** Principal component analysis (PCA) of metabolomics. **(B)** Hierarchical cluster analysis (HCA) of metabolomics. A total of 186 metabolites were measured, revealing that 44 metabolites were changed significantly with endurance exercise. PCA and HCA clearly indicated the difference of metabolic profiles between pre-exercise, post-0 h, and post-6 h.

Plasma metabolomics measured 186 metabolites and revealed that 110 metabolites changed significantly with endurance exercise (Supplementary Table S2). PCA and HCA clearly indicated the difference in metabolic profiles between pre-exercise, post-0 h, and post-6 h (Figure 2).

**FIGURE 3:**
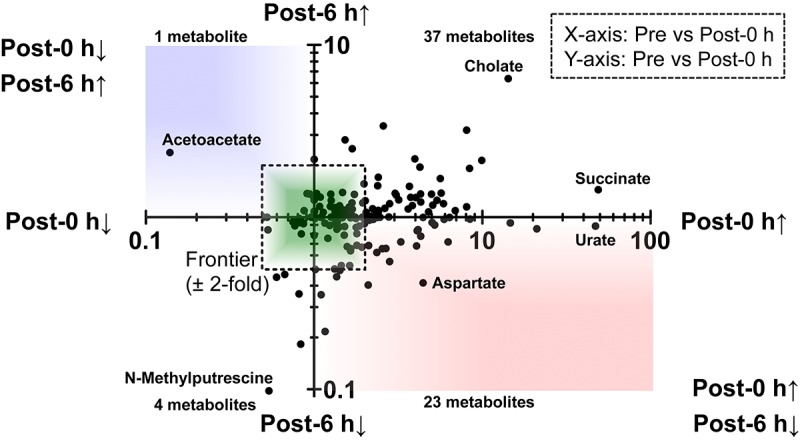
A scatter plot of the fold change of the overlapping metabolites. This figure showed that 23 metabolites, including glycogenic amino acids (such as aspartate, tyrosine, and tryptophan) increased immediately after exercise (post-0 h) and decreased 6 h after exercise (post-6 h) (lower-right quadrant). Also, a metabolite, acetoacetate, decreased immediately after exercise (post-0 h) and increased 6 h after exercise (post-6 h) (upper-left quadrant).

A scatter plot of the fold change of the overlapping metabolites in each condition was generated (Figure 3). This figure shows that 23 metabolites, including glycogenic amino acids (such as aspartate, tyrosine, and tryptophan), increased immediately after exercise (post-0 h) and were decreased 6 h after exercise (post-6 h). Additionally, a metabolite, acetoacetate, decreased immediately after exercise (post-0 h) and increased 6 h after exercise (post-6 h).

### Correlation Between Biomarker Candidates and Brain Glycogen Levels

**FIGURE 4:**
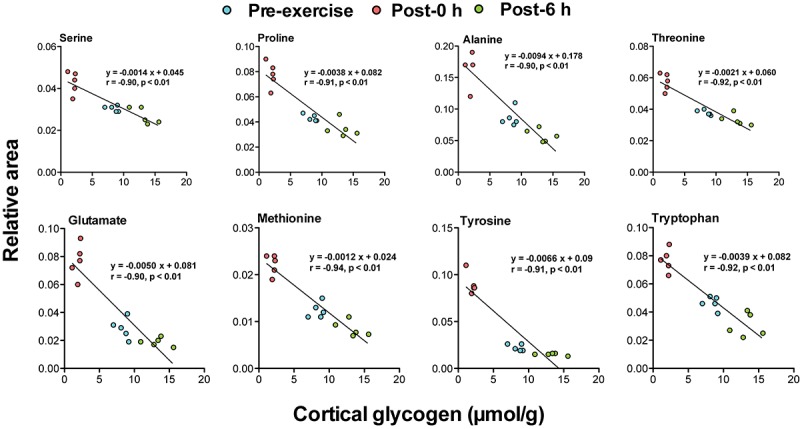
Plasma glycogenic amino acids are associated with cortical glycogen levels during and following endurance exercise. Lines in scatter plots show significant correlation (*P* < 0.05) (Pearson’s product-moment correlations test).

**FIGURE 5:**
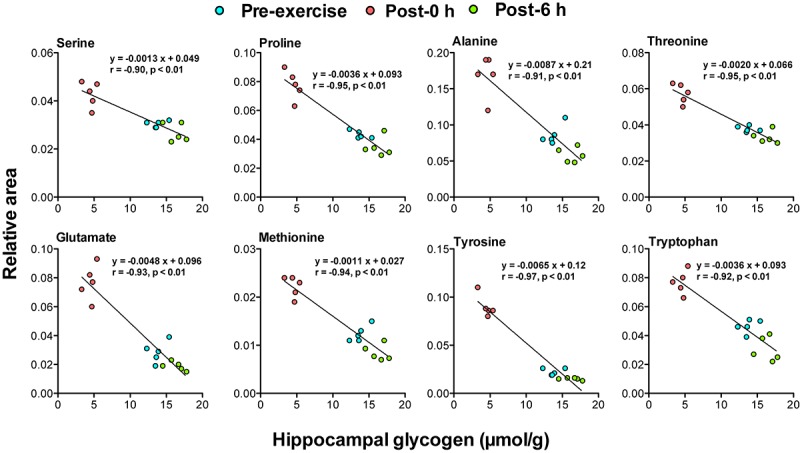
Plasma glycogenic amino acids are associated with hippocampal glycogen levels during and following endurance exercise. Lines in scatter plots show significant correlation (*P* < 0.05) (Pearson’s product-moment correlations test).

**FIGURE 6:**
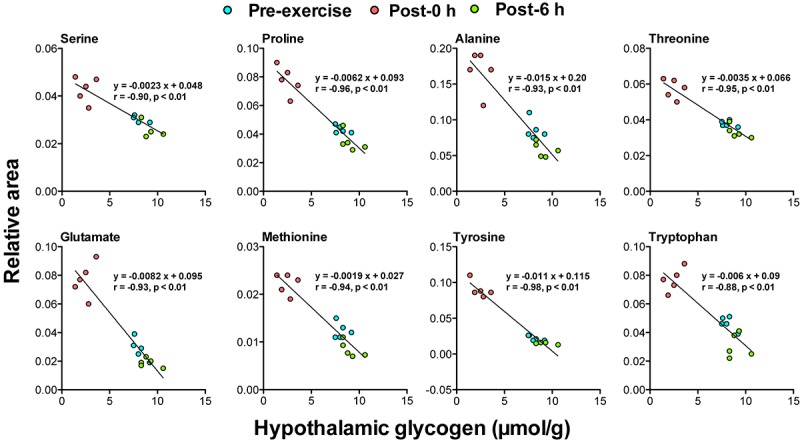
Plasma glycogenic amino acids are associated with hypothalamic glycogen levels during and following endurance exercise. Lines in scatter plots show significant correlation (*P* < 0.05) (Pearson’s product-moment correlations test).

Correlation analysis between 24 plasma biomarker candidates, including glycogenic amino acids and acetoacetate, and brain glycogen levels in the cortex, hippocampus, and the hypothalamus showed that all candidate metabolites were significantly correlated (*P* < 0.05). Furthermore, glycogenic amino acids (serine, proline, threonine, glutamate, methionine, tyrosine, and tryptophan) showed stronger correlations (*r* < −0.9, *P* < 0.05) (Figure 4–6).

## Discussion

This study tested the hypothesis that blood tyrosine is a mechanistic-based biomarker that predicts a decrease and supercompensation of brain glycogen with acute endurance exercise. We first reproduced a rat model of endurance exercise to induce a decrease and supercompensation of brain glycogen (Figure 1). Our metabolomics of plasma samples from rats, that underwent endurance exercise, showed that plasma glycogenic amino acids, including tyrosine, were increased during exercise and were decreased following exercise associated with brain glycogen dynamics (Figure 2–6). These findings support our present hypothesis.

We confirmed hypoglycemia, blood lactate elevation, muscle glycogen depletion, and brain glycogen decrease due to exercise, in the post-0 h group (Figure 1A–C). These phenomena are fatig factors that have been reported by previous studies on prolonged exercise in rodents and humans (Gollnick et al., 1974; Nybo and Secher, 2004; Matsui et al., 2011, 2012), indicating the validity of our rat model for acute endurance exercise. During moderate intensity of endurance exercise, glycogen levels in type II fibers or the plantaris muscle, which consists of over 90% type II fibers, are depleted, similar to that observed in the present study, in rats (Armstrong et al., 1974; Bracken et al., 1988; Matsui et al., 2011, 2012, 2017) and in humans (Gollnick et al., 1974; Vollestad et al., 1984). These previous studies indicate the validity of our glycogen detection. In addition, the decreased brain glycogen due to endurance exercise recovered to higher levels than the basal line within 6 h after exercise, which occurred earlier than muscle glycogen replenishment and supercompensation (Figure 1C), reproducing the onset of brain glycogen supercompensation after endurance exercise found in our previous study (Matsui et al., 2012).

Plasma metabolomics clearly indicated the difference in metabolic profiles among pre-exercise, post-0 h, and post-6 h (Figure 2 and Supplementary Table S2), and glycogenic amino acids, such as aspartate, alanine, tyrosine, and tryptophan, increased immediately after exercise (post-0 h) and decreased 6 h after exercise (post-6 h), which implies that there is a repulsive interaction between brain glycogen dynamics and endurance exercise (Figure 3). Endurance exercise increases blood glycogenic amino acids levels, such as alanine, tyrosine, and phenylalanine derived from protein catabolism in active muscles (Ahlborg et al., 1974), and enhances their splanchnic exchanges to be utilized as hepatic gluconeogenesis sources (Wahren et al., 1971). Furthermore, increased blood glycogenic amino acids recover to basal levels or decrease, compared with pre-exercise levels, to be metabolized by the rest after endurance exercise (Borgenvik et al., 2012; de Godoy et al., 2014). Our present data regarding endurance exercise are supported by these previous findings.

The negative correlations between plasma glycogenic amino acids (serine, proline, threonine, glutamate, methionine, tyrosine, and tryptophan) and brain glycogen levels in the cortex, hippocampus, and hypothalamus were observed (*r* < −0.8, *P* < 0.05) (Figure 4–6). These results indicate, for the first time, the possibility that plasma glycogenic amino acids are biomarkers that predict the decrease and supercompensation of brain glycogen with acute endurance exercise.

**FIGURE 7:**
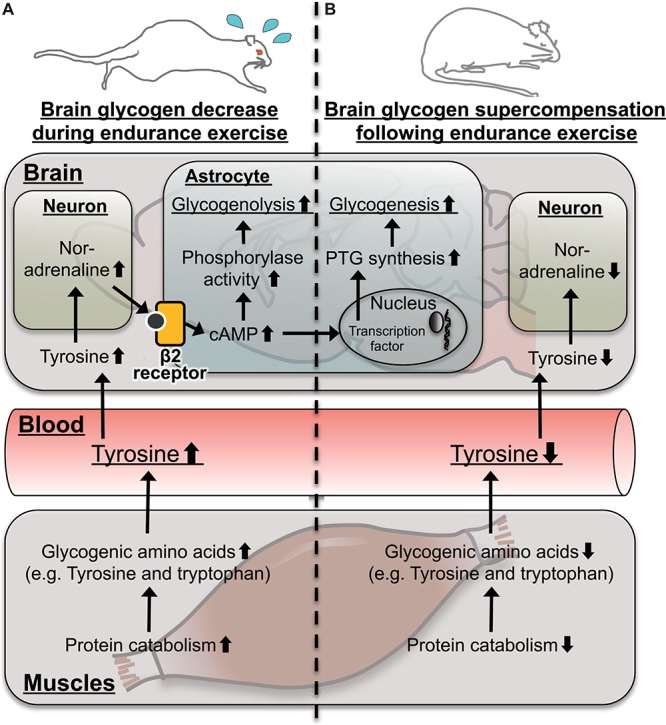
Hypothetical schema for tyrosine as a possible mechanistic-based biomarker predicting brain glycogen dynamics **(A)** during and **(B)** following endurance exercise. In the skeletal muscle, protein catabolism is activated to produce glycogenic amino acids including tyrosine. Tyrosine is released to the blood stream, and its level is increased. Blood tyrosine is taken up by the brain and is converted into noradrenaline in noradrenergic neurons. Noradrenaline activates cyclic adenosine monophosphate (cAMP) production via the β2-adrenaline receptor to activate glycogenolysis in a matter of minutes but takes hours to induce glycogenesis and supercompensation through expression of protein targeting to glycogen (PTG). This late onset of glycogen synthesis likely contributes to supercompensation following endurance exercise.

In particular, tyrosine can be a valuable mechanistic-based biomarker for brain glycogen dynamics with endurance exercise, a concept which is shown in Figure 7. In active skeletal muscles during endurance exercise, levels of glycogenic amino acids are increased through protein catabolism (Ahlborg et al., 1974). The increases in levels of blood glycogenic amino acids, including tyrosine, are derived from muscles (Ahlborg et al., 1974). Increased blood tyrosine, a precursor of noradrenaline, is taken up by noradrenergic neurons in the brain (Hufner et al., 2015; Alabsi et al., 2016; Imai et al., 2017). Noradrenergic neurons release noradrenaline into the intracellular fluid, and the noradrenaline activates glycogenolysis through cAMP production mediated by the β2 receptor in the astrocytes (Magistretti et al., 1981; Magistretti, 1988). Actually, endurance exercise decreases glycogen levels associated with activated noradrenergic turnover in the cortex (Matsui et al., 2011). Therefore, tyrosine is a possible biomarker for brain glycogen decrease during endurance exercise (Figure 7A).

Furthermore, noradrenaline activates not only glycogenolysis but also glycogenesis and supercompensation through the expression of PTG, mediated by cAMP in astrocytes (Sorg and Magistretti, 1992; Allaman et al., 2000; Ruchti et al., 2016). Following endurance exercise, while glycogen synthesis can be activated through PTG, glycogenolysis is not active, due to decreased brain noradrenaline along with blood tyrosine, and as a result, brain glycogen supercompensation is likely induced. Serotonin, which is synthesized from tryptophan in brains, also activates astrocytic glycogenolysis through a Ca^2+^ influx (Gibbs and Hertz, 2014), but there is no report for PTG induction. Glutamate activates glucose uptake in astrocytes but does not play a direct role in glycogen metabolism (Hamai et al., 1999). Thus, plasma tyrosine, rather than tryptophan and/or glutamate, is a possible biomarker not only of a brain glycogen decrease during endurance exercise but also of brain glycogen supercompensation following endurance exercise (Figure 7B).

Furthermore, plasma levels of branched chain amino acids (BCAA) such as leucine, isoleucine, and valine were not significantly changed by endurance exercise (Supplementary Table S2). Large-neutral amino acids such as leucine, isoleucine, valine, phenylalanine, tryptophan, and tyrosine share the same transporter, L-type amino acid transporter 1 (LAT1), on the blood-brain barrier (BBB) (Pardridge and Oldendorf, 1977; Boado et al., 1999). Since BCAA levels were unchanged, the LAT1 at the BBB would be ready for use by other neutral amino acids such as tyrosine, which increased in the plasma during exercise.

The use of an acute endurance exercise model in rodents produced new evidence on plasma tyrosine as a mechanistic-based biomarker for brain glycogen dynamics in endurance exercise. In this study, however, glycogen levels after post-6 h was not examined. Therefore, although chronic exercise or brain glycogen loading increases brain glycogen levels in the cortex, hippocampus, and the hypothalamus (Matsui et al., 2012; Soya et al., 2018), it is still unclear whether tyrosine is a useful biomarker not only for acute endurance exercise but also for chronic exercise or glycogen loading. Furthermore, here, we tried to investigate the brain region specificity of biomarker candidates, but it was not revealed because glycogenic amino acids correlated strongly with glycogen levels in all brain loci we detected in the present study (the cortex, hippocampus, and hypothalamus). These important issues should be addressed in future research.

In conclusion, our metabolomics of plasma samples from rats showed quantitative differences in glycogenic amino acids, during and following endurance exercise. In particular, plasma tyrosine, a precursor of noradrenaline, is a possible mechanistic-based biomarker for brain glycogen dynamics during and following endurance exercise. These findings support our present hypothesis that blood tyrosine is a mechanistic-based biomarker that predicts a decrease and supercompensation of brain glycogen with acute endurance exercise. Plasma tyrosine may contribute to the development of a valuable parameter for exercises as a training/conditioning for athletes and/or therapeutic strategy for neurodegenerative diseases.

## Data Availability

All datasets generated for this study are included in the manuscript and/or the Supplementary Files.

## Ethics Statement

All experimental protocols were approved by the Institutional Animal Care and Use Committee of the University of Tsukuba, and all procedures and methods were performed in accordance with the relevant guidelines laid down by the animal ethics committee (Animal ethical approval number; 15–055). Every effort was made to minimize the number of animals used as well as any pain and discomfort.

## Author Contributions

TM and HS conceived and designed the experiments. TM, Y-FL, MS, and TS collected the data. TM, Y-FL, MS, TS, and HS analyzed and interpreted the data. TM and HS drafted the manuscript and revised it critically for important intellectual content. All authors approved the final version of the manuscript.

## Conflict of Interest Statement

The authors declare that the research was conducted in the absence of any commercial or financial relationships that could be construed as a potential conflict of interest.
