# Supplementary material for: Tyrosine as a Mechanistic-Based Biomarker for Brain Glycogen Decrease and Supercompensation With Endurance Exercise in Rats: A Metabolomics Study of Plasma
Source: Front Neurosci. 2019 Mar 19;13:200. doi: 10.3389/fnins.2019.00200 (PMC6433992; doi:10.3389/fnins.2019.00200)
Supplement: Supplementary file 1 [file Table_1.pdf]

Supplementary table 1 Habituation protocol for treadmill running exercise.

Day	Running speed and time
1	Rest, 10 min + 5 m/min, 10 min + 10 m/min, 10 min
2	Rest, 5 min + 5 m/min, 10 min + 10 m/min, 10 min + 15 m/min, 10 min
3	Rest
4	Rest, 5 min + 10 m/min, 10 min + 15 m/min, 10 min + 20 m/min, 10 min
5	Rest, 5 min + 15 m/min, 10 min + 20 m/min, 10 min + 25 m/min, 10 min
6	Rest, 5 min + 15 m/min, 10 min + 20 m/min, 10 min + 25 m/min, 10 min
